# Assessment of Upper Limb Movement Impairments after Stroke Using Wearable Inertial Sensing

**DOI:** 10.3390/s20174770

**Published:** 2020-08-24

**Authors:** Anne Schwarz, Miguel M. C. Bhagubai, Gerjan Wolterink, Jeremia P. O. Held, Andreas R. Luft, Peter H. Veltink

**Affiliations:** 1 Biomedical Signals and Systems (BSS), University of Twente, 7500 AE Enschede, The Netherlands; miguel.bhagubai12@gmail.com (M.M.C.B.); gerjan.wolterink@utwente.nl (G.W.); p.h.veltink@utwente.nl (P.H.V.); 2 Vascular Neurology and Neurorehabilitation, Department of Neurology, University Hospital Zurich, University of Zurich, 8091 Zurich, Switzerland; jeremia.held@usz.ch (J.P.O.H.); andreas.Luft@usz.ch (A.R.L.); 3 Robotics and Mechatronics group, University of Twente, 7500 AE Enschede, The Netherlands; 4 Cereneo, Center for Neurology and Rehabilitation, 6354 Vitznau, Switzerland

**Keywords:** upper extremity, stroke, biomechanical phenomena, kinematics, inertial measurement systems, motion analysis

## Abstract

Precise and objective assessments of upper limb movement quality after strokes in functional task conditions are an important prerequisite to improve understanding of the pathophysiology of movement deficits and to prove the effectiveness of interventions. Herein, a wearable inertial sensing system was used to capture movements from the fingers to the trunk in 10 chronic stroke subjects when performing reach-to-grasp activities with the affected and non-affected upper limb. It was investigated whether the factors, tested arm, object weight, and target height, affect the expressions of range of motion in trunk compensation and flexion-extension of the elbow, wrist, and finger during object displacement. The relationship between these metrics and clinically measured impairment was explored. Nine subjects were included in the analysis, as one had to be excluded due to defective data. The tested arm and target height showed strong effects on all metrics, while an increased object weight showed effects on trunk compensation. High inter- and intrasubject variability was found in all metrics without clear relationships to clinical measures. Relating all metrics to each other resulted in significant negative correlations between trunk compensation and elbow flexion-extension in the affected arm. The findings support the clinical usability of sensor-based motion analysis.

## 1. Introduction

Human hand and arm function contribute to a wide range of activities in daily life, ranging from sensory functions to interacting with the environment and to functions that have a strong motor component like the manipulation of objects in grasping [1]. Hand and arm functionalities including object manipulation and physical interactions with the environment rely on the ability to control prehensile finger forces to perform specific grasp types [2,3] and ability to control both the distal and proximal joints of the upper limb in a goal-directed manner [4], for example when transporting the hand to reach the location of a desired object and forming the fingers for grasping [5].

In subjects, experiencing upper limb impairments due to a stroke, these complex hand- and arm-grasping functionalities are defective [6]. Stroke is known as the leading cause of disability in the world [7], defined as a disruption in brain cell perfusion that leads to cell death and losses in network connectivity and multimodal impairments [8]. In particular, infarctions of the middle cerebral artery affecting the primary motor cortex and the integrity of the corticospinal tract have been associated with upper limb movement deficits [9,10], such as weakness, decreased interjoint coordination and in particular diminished finger dexterity [11,12]. Of these motor performance aspects, weakness caused by stroke indicates the inability to activate certain upper limb muscles or segments, whereas interjoint coordination is defined as the ability to control all upper limb joints or segments in a spatially and temporally efficient manner. The differentiation between weakness and interjoint coordination during upper limb activities is rather precisely definable, as both show strong associations with each other [13] and other stroke-related impairments, such as spasticity [14]. Phenotypes of stroke-related interjoint coordination deficits include the appearance of the pathological flexor synergy in reaching, increased trunk movements to compensate for the upper limb limitations and a decreased finger dexterity for prehensile grasp application. The pathological flexor synergy was defined as a stereotypical co-activation of elbow flexion and shoulder abduction [15] that becomes visible in reaching [14], in arm-load related reductions in upper limb workspace [16], and in a diminished ability to extend the fingers [17,18]. Engaging trunk movements in reaching has been considered as movement strategies to compensate for upper limb motor impairments with associations to the level of impairment [19]. These stroke-related movement abnormalities might become present in isolation or combination and chronically manifested depending on the severity of deficit and cerebral region affected [11], thereby presenting a continuous challenge for treatment approaches.

Being able to capture these upper limb movement characteristics is important to improve the understanding of stroke-related movement deficits, including their possible underlying dysfunctions, and to further investigate effectiveness of approaches to influence these deficits [20]. In this regard, it needs to be considered that upper limb movements can be assessed on different levels. Approaches to evaluate and assess upper limb movement deficits after strokes range from more or less extensive qualitative descriptions of body functions and activities in therapeutic records of clinical practice, over clinical scales that mostly rely on observer-based scoring and time-efficiency measures, to instrumentations and technologies for kinematic motion analysis. Although clinical assessments, such as the Fugl–Meyer Assessment of the Upper Extremity (FMA-UE) or the Action Research Arm Test (ARAT), have demonstrated excellent reliability and validity as assessment tools [21], their level of information detail, mostly due to the gross ordinal scoring nature of relatively complex defined movement items, does not allow to sufficiently discriminate physiological and above-mentioned pathological movement behavior [20].

Kinematic assessments on the other hand are supposed to offer fine-grained and objective outcomes on movement quality and have shown to detect stroke-related movement impairments in terms of longer movement times, greater trunk displacement and less elbow extension in reaching movements [22,23]. However, the widespread application of kinematic measurements in clinical practice faces several barriers. First, the high variety of measurement systems with different considerations on interaction forces, movement tasks and different metric derivations hampers the comparability and conclusion drawing [24]. Secondly, investigations of complete motion kinematics including trunk and finger motions were sparse. Thirdly, most of the measurement systems being used were optoelectronic and robotic systems that are based on fixed laboratory environments and expensive equipment [25]. Being able to perform comprehensive upper-limb kinematic analysis outside of the laboratory, in flexible environments with the least possible influence on movement behavior would facilitate implementation of kinematic measurements of qualitative aspects movement behavior in clinical practice. In setting up this pilot study, it was aimed to address the outlined limitations by extensively measuring and quantifying reach-to-grasp movements after stroke, with respect to interjoint coordination determined by trunk compensation and flexion-extension of the elbow, wrist and fingers, specifically quantified during active grasp and object displacement. A portable inertial system was used to measure complete upper limb kinematics, from the trunk to the fingertip, including fingertip force sensing in flexible experimental tasks and set-up environments. Different task characteristics, such as the target locations and the object to be grasped were considered in the experimental design to investigate influences of additional arm load and workspace relations including increased mechanical work demands in movements against gravity on metrics for determining inter-joint coordination. It is assumed that the reaching movement might result in different joint executions with respect to different target locations in the workspace, e.g., features of the pathological flexor pathology might become more pronounced in target positions with higher anti-gravitational mechanical work and with more distance from the body center. Likewise, grasping different object weights results in different additional armloads, that could affect the ability to perform unaffected reaching.

The primary study goal was to evaluate spatiotemporal kinematic metrics for the assessment of upper limb movements after stroke. It was first questioned whether changes in the kinematic range of motion (ROM) in terms of joint angle ranges can be attributed to the factor tested arm, object weight and target height during object displacement. The second question was how far the kinematic metrics relate to clinically measured upper limb impairment. The third question related to the correlation between each of the joint range metrics to evaluate potential joint coupling, such as the pathological flexor synergy between shoulder flexion-extension, elbow flexion-extension and trunk compensation.

## 2. Materials and Methods

### 2.1. Study Design and Participants

This pilot study was set up to investigate upper limb motion primitives from proximal to distal function in stroke subjects by use of a wearable inertial sensing system. The study was approved by cantonal ethics in Zurich (BASEC-No: Req-2019-00417) and carried out in accordance with the declaration of Helsinki. Subjects after stroke were recruited from a University Hospital Zurich Stroke Registry and invited for a single-session measurement of two hours at the Clinic of Neurology of the University Hospital Zurich, Switzerland.

Subjects were included if they were at least 18 years old, able to give informed consent and had been diagnosed with unilateral stroke at least six months before the study onset with associated upper limb impairments. Subjects had to have at least partial ability to move the arm against gravity and to perform finger movements for basic grasp function. Exclusion criteria were pre-existing deficits of the upper limb, such as orthopedic impairments, severely increased muscle tone with limitation in range of motion in the upper limb (Modified Ashworth Scale of >2 in one of the upper limb muscle groups), severe sensory deficits in the upper limb (absence of light touch in the hand and fingers), and severe communication or cognitive deficits that cause inability to follow the procedures. Participant characteristics of interest included the gender, age, stroke location side, time since stroke, stroke affected cerebral perfusion territory and the severity of upper limb motor impairment, as measured with FMA-UE. The FMA-UE is a cumulative numerical scoring system to evaluate motor function after stroke, which consists of an arm, wrist, hand and coordination subsection to account for independent severity and recovery patterns, presented in a full score range from 0 to 66 points [26].

### 2.2. Measurement System

**Figure 1:**
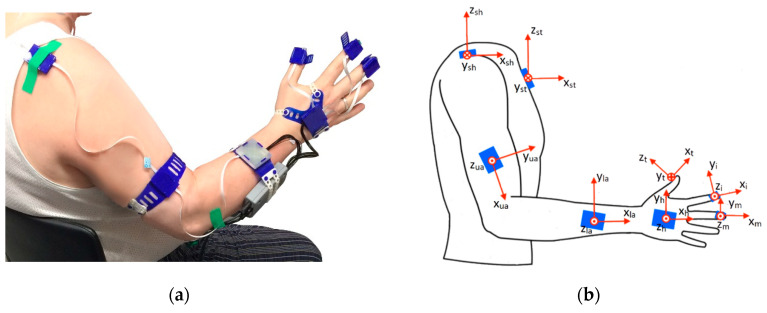
Wearable inertial sensing system: (**a**) system set-up; (**b**) anatomical frame definition per segment.

The wearable inertial sensing measurement system was a modified version of the inertial measurement unit (IMU)-based hand and finger sensing system, reported and evaluated by Kortier et al. [27]. It was composed of eight IMUs, with triaxial accelerometers and gyroscopes, based on a micro-controller-based sensing system principles of the PowerGlove [27,28] that were covered by 3D-printed housings, and combined with force sensors. The IMUs were placed and fixated at the sternum, shoulder, upper arm, lower arm, hand, thumb, index and fingers with medical tape or 3D-printed flexible straps (Figure 1). Additionally, the finger IMUs were combined with force-sensitive resistors (FSR) to detect interaction forces between the object to be grasped or manipulated and the finger pad. The upper arm IMU was placed on the lateral side of the arm, close to the elbow and the lower arm IMU was placed on the dorsal side of the forearm, close to the wrist. The hand sensor was placed on the back of the hand, and the thumb, index and middle finger IMUs were attached on the fingertips of the respective fingers, with the force-sensitive resistors fixed on the finger pad by the IMU housing’s strap. Each pair of triaxial accelerometers and gyroscopes (ST LSM330DLC manufactured by STMicroelectronics, Geneva, Switzerland) were contained within small printed circuit boards (PCBs). The separately encased IMUs were connected via flexible cabling strips, forming two separate strings (arm string—containing the sternum, shoulder, upper and lower arm sensors; and hand string—containing the hand and fingers sensors). Signals from both sensor strings were collected and connected through a bus master/microcontroller (Atmel XMEGA manufactured by Atmel, California, USA) and streamed real-time via the USB channel onto a PC for control in Matlab software (MATLAB version 2016b, The Mathwork, Natick, MA, USA). Acceleration data was collected with a sampling frequency of 100Hz and gyroscope data with a frequency of 200 Hz. Both were low-pass filtered by using a Butterworth filter with a cut-off frequency of 10 Hz

### 2.3. Kinematic Reconstruction

All sensors were calibrated each day prior to the measurements by placing them inside a box with orthogonal sides, which was turned over 90 degrees in all three orthogonal directions. The accelerometer bias in the different axes and the gyroscope static bias was measured before the whole experiment per subject and compensated during the measurements [29]. The kinematic reconstruction was based on the estimation of the sensors’ orientation, which is taken from the acceleration and angular velocity measures of the IMUs. In order to estimate the orientation of the limb segments, a sensor-to-segment calibration was performed, as well as a definition of a common global frame for all sensors. The sensor-to-segment calibration was carried out to determine the upper body anatomical axes of the limb segments (joints) relative to the corresponding sensors by performing ten different postures and movements that were based on Luinge et al. [30] and Ricci et al. [31]. The equipped test person was assisted by a trained research clinician to perform the calibration protocol, which consisted in eight static positions and two dynamic movements, as shown in Table 1.

In the static positions, the gravity vector measured by the accelerometers represents one of the axes. In the dynamic movements, the angular velocity, depending on the rotation direction, also represents the rotation around a specific anatomical axis of a body segment. For each anatomical frame, two different axes were measured using either the accelerometer or gyroscope, depending on the segment. The third axis is calculated using the cross-product of the previous two axes. Subsequently, an orthonormal coordinate axis was based on these three axes. The last two movements, standing straight and bending by hip flexion, were used to determine the global frame and initial sensor orientation estimation [32]. The static neutral pose, with the arm stretched along the body and the fingers extended, gives the common vertical axis by measuring the gravity vector in all sensors. The hip flexion movement is performed with the arms extended along the body for the definition of the horizontal axis of the global frame. With the sensor-to-segment alignment and the common global frame for every IMU, it is possible to reconstruct the movement of the trunk, arm, hand, and fingers. Integration drift of the angular velocity over time was corrected by applying a Madgwick filter to correct for the inclination error of the sensor with respect to the gravitational component of the accelerometers [33]. Drifts in the gyroscope orientation were reduced by zero-velocity updates, following the methods of Kirking et al. [34] where if the norm of the angular velocity is below 3°/s is defined to be static in terms of actual sensor movements.

The joint angles are defined as the angle between two anatomical axes of adjacent limb segments of the respective joint as indicated in Figure 1b. Positive angles indicate flexion, abduction or supination of a joint and a negative angle indicates extension, adduction or pronation.

Three trunk compensation angles were calculated by comparing the projected trunk axes onto the global frames corresponding to the static neutral pose, consisting of trunk flexion (rotation in the sagittal plane around the *y*-axis of the sternum), lateral rotation (rotation around the *x*-axis of the sternum), and torsion (rotation around the *z*-axis of the sternum). *Shoulder flexion/extension* was defined as the angular variation of the upper arm’s x-axis ($x_{ua}$) in the frontal plane (defined by the *x*-*z* plane of the sternum’s frame). Shoulder abduction/adduction is determined by relating the upper arm ($x_{ua}$) to the sternum’s frame in the frontal plane (defined by the *y*-*z* plane of the sternum, see Figure 1b). Elbow flexion/extension was determined by the angle between the upper ($x_{ua}$) and the lower arm’s ($x_{la}$) *x*-axis. Forearm supination/pronation was defined by the mean orientation variation around the *x*-axis of the lower arm ($x_{la}$). Wrist flexion/extension was defined by the angle between the *x*-axis of the lower arm ($x_{la}$) and the hand’s *x*-axis ($x_{h}$). The finger flexion/extension (thumb, index finger and middle finger) was defined as the angle between the *x*-axis of the hand ($x_{h}$) and the fingertip frames ($x_{m}$, $x_{i}$ and $x_{t}$).

### 2.4. Experimental Protocol

**Figure 2:**
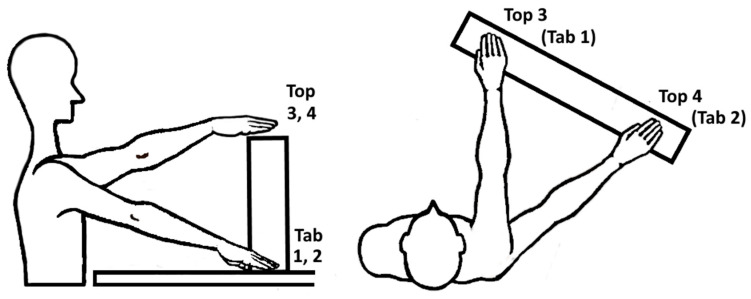
Experimental set up in sagittal and top view including the target locations: Tab 1; in ipsilateral arm length, Tab 2; in abducted arm length, Top 3; ipsilateral arm length, Top 4; in abducted arm length. Block objects: BL (big light block, 108 g), BW (big wooden block, 490 g) and BH (big heavy block, 1008 g).

At the beginning, each participant was interviewed about demographic information and assessed for upper limb impairments by use of the FMA-UE [26]. The experimental protocol was performed on both limbs separately, starting with the non-affected limb (NAF) followed by the affected limb (AF), to study differences between pathological and physiological movement behavior. The protocol consisted in performing reach-to-grasp and displace different types of cubic blocks to different target positions. The participant was positioned sitting in front of a table with the tested arm held in 90° elbow flexion and the palm facing down on the table. Three markers were defined on the table for placing the hand and fingers in the starting position. The target positions were determined by each participants’ maximal arm length in four pre-defined target locations as shown in Figure 2 and mirrored between both upper limbs. This task set-up was adapted from the ARAT [35], which evaluates the ability to grasp and displace, for example wooden blocks, onto a 37 cm high to-shelf.

The target locations at table height (1, ipsilateral arm length and 2, abducted arm length) and at top-shelf height (3, ipsilateral arm length and 4, abducted arm length) were selected to explore kinematic expressions in a relevant arm workspace and observe effects of arm loading in movements against gravity. The 10 cm block objects to be grasped varied in three different weights: 108 g (BL, big light block), 490 g (BW, big wooden block) and 1008 g (BH, big heavy block) to investigate influence of additional load during object grasp and displacement. The weight range of the object was based on the weight of the standardized weight of the wooden block (490 g) that is used in the Action Research Arm Test. The 1kg weight was selected as it corresponds to objects, relevant for daily-life functioning daily life, e.g., when manipulating a 1 l bottle of water. The lighter block was included to enable the movement analysis with only little additional weight load. The order of blocks was randomized in advance to avoid task-related physical fatigue during the experiments. This resulted in a combination of 12 task conditions per tested arm, that were each repeated three times.

After donning the system, the sensor-to-segment calibration protocol was performed with manual guidance of a therapist to assure proper execution of the static positions and the dynamic movements. Each position was measured for at least five seconds and checked online by an experienced engineer. For accurate global frame definition and sensor orientation estimations, the last two calibration movements were performed before each measurement trial. This procedure allowed to reduce drift in the sensor data during measurements. In between the three repetitions, each subject was asked to avoid extra movements of the tested arm and go back to the starting position as soon as the movement task was finished. This procedure allowed the subjects to rest for about 10 s between the trials. The whole experiment was expected to be performed within a maximum of 2 h. After the system was donned, each participant rated the wearing comfort of the system, possible limitations of gross movements due to the cables and limitations of grasping due to the fingertip sensors on a 5-point Likert-scale.

### 2.5. Feature Extraction

**Figure 3:**
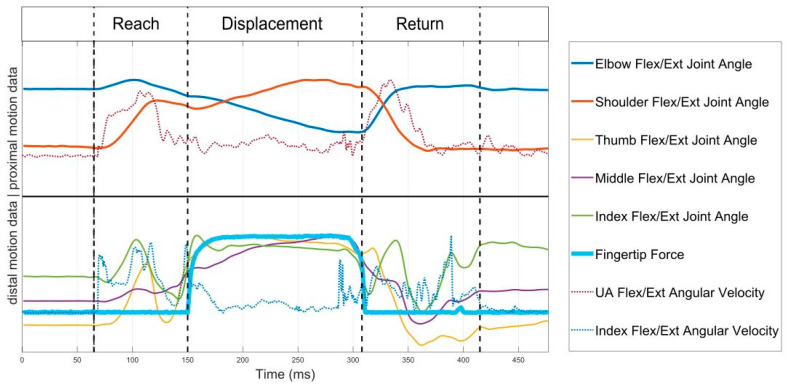
Proximal (shoulder, elbow), distal (finger) motion data and force signal for phase segmentation. The data is scaled to fit the plot, not the actual measured values on the *y*-axis.

To enable task-specific spatiotemporal analysis of the reach-to-grasp movements, each movement trial was segmented into three phases: (1) reach, (2) displacement and (3) return by determining the time points of movement onset, grasp, release and movement end. Movement start and movement end were detected using a threshold detection algorithm for the upper arm’s IMU angular velocity norm, where a threshold of 0.1 rad/s was used [36] to account for relevant limb motion. The force data of the fingertips on the table was also used as an onset and offset indicator, where applicable. The moment of grasping was defined by the detection of finger reaction and interaction forces, whereas the release is defined by the decrease in force signal to the lowest value of force, as displayed in Figure 3. In cases where no force profile was detected due to low interaction forces or because the finger contact points deviating from the force sensor placements, the grasp and release time points were identified via the joint angle profiles. The release time point was defined by the changes from finger flexion to extension including the maximum elbow extension and shoulder flexion, that represent the moment of maximal reach to target position. The duration of each movement phase was calculated as the time between the delimitating time points of each phase.

For validation of the relevant expected differences between physiological and pathological movement behavior in the study sample, movement time and active range of motion of the main degrees of freedom (DOF) were compared between the affected and non-affected side. Movement time was defined as the time between movement start and end, detected by the 0.1 rad/s threshold. The DOF included trunk compensation, shoulder flexion-extension, shoulder abduction-adduction, elbow flexion-extension, forearm supination-pronation, wrist flexion-extension, thumb, middle finger and index finger flexion-extension for the entire task analysis per target location of the reach-to-grasp movement.

The primary outcome parameters, range of motion in trunk displacement, elbow, wrist, and finger flexion-extension were defined as the difference between the maximum and minimum angle of the joint during the period of object displacement, because they were expected to show expressions of the pathological flexor synergy and compensatory trunk movements. Kinematic parameters of interest to determine interjoint-coordination during the reach-to-grasp movement were defined as the joint ranges in trunk displacement, elbow, wrist, and finger flexion-extension within the displacement phase of the task. Trunk compensation in degrees was used as a metric to quantify the amount of compensatory trunk inclination during the upper limb movement and was defined by the square root of the sum of squares of the ranges in all three trunk compensation angles. Range of motion in elbow and wrist flexion-extension were calculated by taking the difference between the maximum and minimum joint angle measured in the displacement phase, as a metric for quantifying the pathological flexor synergy. The range of motion in finger flexion-extension was calculated as the mean between the range of the index and the middle fingers for each movement execution to consider distal characteristics of the pathological flexor synergy.

#### Statistical Analysis

All outcome parameters were visually inspected in histograms and presented descriptively by means and standard deviations.

Differences in range of motion in trunk displacement, elbow, wrist, and finger flexion-extension during object displacement were analyzed with respect to tested arm, object weight and target height, by considering the average of the three repetitions per subject and task condition. A linear mixed model analysis was applied to investigate significant differences and interactions between the independent factors, arm (AF, NAF), object (BL, BW, BH) and target height (Tab, Top), on the dependent variable of the metrics on joint range of motion, as presented in the model: Metric−a1 × Arm + a2 × Weight + a3 × Height + a4 × Subject. The linear mixed model analysis was selected as it takes into account the repeated measures experimental design and inner subject effects in a nested structure of the dependent variables.

The analysis of the relationship between the displacement phase kinematics of trunk displacement, elbow, wrist, and finger flexion-extension and the individual impairment level, as determined with the clinical FMA-UE test, was explored by plotting the median joint ranges including the upper and lower boundaries of the interquartile range of the affected arm against the measured impairment with the FMA-UE. Statistical testing for answering the second and third research question was performed by cross correlations based on Spearman rank correlations to investigate the relationships between FMA-UE, trunk displacement, elbow flexion-extension, wrist flexion-extension, and finger flexion-extension. All statistical tests were performed using Matlab (MATLAB version 2016b, The Mathwork, Natick, MA, USA) and SPSS (SPSS version 26.0, IBM Corp., Armonk, NY, USA) with a significance level of *p* = 0.05, indicating significances of *p* = 0.01 and *p* = 0.001 specifically.

## 3. Results

Kinematic measurements were gathered in 10 chronic stroke subjects within a recruitment period of 8 days in July 2019. One subject performed only two of the three block conditions due to time constraints. The data of the remaining blocks were discarded due to incomplete and incorrect sensor-to-segment calibration data. This resulted in a total of nine out of 10 subjects, who were included in the data analysis, adding up to 324 affected and non-affected side motion data sets. All participants rated the measurement system to be comfortable to wear. One subject rated some influence on the gross movements due to the cable wires of the sensing system. Three of the participants reported impedance of grasp due to the finger sensors.

### 3.1. Demographics

The demographics of the study participants are shown in Table 2, consisting of four right-side dominant and five left-side affected subjects. Upper limb impairments were measured with the FMA-UE score, ranging from 28 to 46 out of 66 points. Subjects with strokes in the perfusion territory of the middle and posterior cerebral artery showed slight increased upper limb impairments (FMA-UE mean 32.6) when compared to those with strokes in the anterior cerebral artery area (FMA-UE mean 43). According to a group analysis of the upper limb capacity-levels in relation to FMA-UE score [37], this sample included one subject with poor capacity (FMA-UE 23–31), eight subjects showing limited capacity (FMA-UE 32–47) and no subject with notable capacity (FMA-UE 48–52) or full function (FMA-UE 53–66).

### 3.2. Upper Limb Kinematic Measures

The automated detection algorithms were successfully applied in 56.1% of the data. Corrections had to be made in 47.8% of the NAF data and 52.2% of the AF data. Failures in automated detection were 76.5% related to inconsistent or low force profiles and in 23.5% related to jerky and noisy angular velocities or joint angle profiles and manually corrected.

Statistically significant higher movement times were found in the AF (mean 4.9 ± 1.6 s) when compared to the NAF (mean 2.8 ± 5.4 s) for the whole task execution (*p* < 0.000) and accordingly for all subphases (*p* < 0.000) of the reach-to-grasp movement. The mean difference in ROM across the main DOF between the AF and the NAF was 10.0 ± 6.9 degrees across the investigated joints, ranging from 0.2 to 28.7 degrees. The differences in range of motion between the AF and NAF were statistically significant across target locations for shoulder flexion-extension, elbow flexion-extension, wrist supination-pronation, thumb, and index finger flexion-extension, as shown in the Appendix A, Table A1. Range of motion in flexion-extension of the shoulder and the elbow were consistently lower in the AF when compared to the NAF, indicating a limited ability to elevate the arm and extend the elbow in reaching. Trunk compensation was significantly different between AF and NAF for the two abducted target locations. A higher mean flexion-extension range was detected for both the index and the middle finger of the AF compared to the NAF, besides lower flexion-extension ranges in the thumb of the affected side for all target positions.

### 3.3. Influences of the Factors, Arm, Object Weight and Target Height on Joint Range of Motion

For each of the primary kinematic features (trunk compensation, elbow, wrist, and finger flexion/extension), significant differences in range of motion of the displacement phase can be attributed to the factors tested (arm, object weight, target location). The results of estimates for the independent fixed factors arm (affected side vs. non-affected side), object (BL, BW, BH) and target height (table location vs. top location) on the selected DOF are shown in Table 3.

The factor of the tested arm showed significant effects on trunk compensation with larger range of motion in the AF (mean 9.4 ± 1.2 degrees) when compared to the NAF (mean 8.2 ± 1.1 degrees) with *F* = 8.327, *p* = 0.006. Elbow flexion-extension was significantly lower in the AF (mean 44.3 ± 3.9 degrees) than in the NAF (mean 54.2 ± 4.6 degrees) resulting in significant effects of the arm tested with *F* = 23.385, *p* = 0.000. Higher ranges in wrist flexion-extension were found in the AF (mean 29.4 ± 4.2 degrees) than in the NAF (21.2 ± 2.7 degrees) with *F* = 30.798, *p* = 0.000 and in finger flexion-extensions of the AF (mean 99.6 ± 11.4 degrees) when compared to the NAF (mean 77.1 ± 9.0 degrees) with *F* = 29.553, *p* = 0.000.

Significant effects for the fixed factor of object weight were found on the metric of trunk compensation (*F* = 4.238, *p* = 0.022). Considering post-hoc pairwise testing, trunk displacement was significantly larger when displacing the big heavy block (mean 10.2 ± 1.9 degrees) when compared to the displacement of the big light block (mean 7.9 ± 1.4, *p* = 0.026) and non-significantly larger in comparison to the big wooden block (mean 8.4 ± 1.3 degrees, *p* = 0.067).

The factor height showed significant effects on all DOF. Displacement to the top height location resulted in significantly higher trunk compensation (mean 9.5 ± 1.3 degrees) when compared to table locations (mean 8.1 ± 1.1 degrees, *p* = 0.006). The highest statistically significant effect was found in elbow flexion-extension with pronouncedly increased range of motion in the top height location (mean 61.3 ± 4.4 degrees) when compared to the table locations (mean 37.2 ± 3.9 degrees, *p* = 0.000) with *F* = 147.742, *p* = 0.000. Likewise, ranges in wrist flexion-extension and finger flexion were increased in the top locations with a wrist flexion-extension mean of 23.7 ± 3.4 degrees in the table locations when compared to a mean of 26.9 ± 3.8 degrees in the top locations (*F* = 4.354, *p* = 0.040) and a finger flexion-extension mean of 82.1 ± 9.9 degrees in the table locations and a mean of 94.4 ± 11.1 degrees in the table locations (*F* = 7.920, *p* = 0.006).

### 3.4. Relationship between Kinematic Parameters and Clinical Measures of Impairment

**Figure 4:**
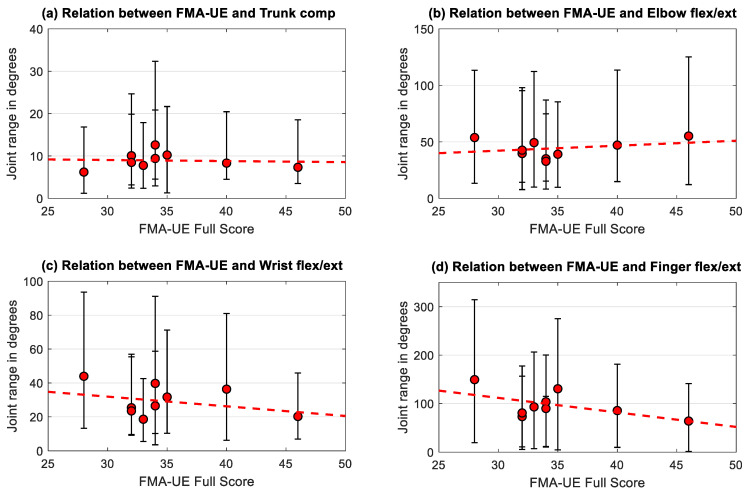
Subjects median joint range of (**a**) trunk compensation, (**b**) elbow, (**c**) wrist, and (**d**) finger flexion/extension of the affected side in relation to impairment level (FMA-UE score ranging from 0–66 points). Error bars represent the interquartile range over all trials performed by each of the nine subjects and the regression lines over the subjects are included for each metric.

For investigating the relationship between the individual participants’ impairment level, as indicated by the FMA-UE score, and the joint ranges of the affected side during displacement, the FMA-UE score was plotted against the subjects median range of motion in trunk compensation and flexion-extension of the elbow, wrist, and fingers as visualized in Figure 4a–d. Three repetitions, three block weights and four target positions were considered for each subject resulting in 36 trials per subject and tested arm, represented by median and interquartile range. There was no significant correlations found between the FMA-UE and the individuals mean trunk compensation (r = 0.11, *p* = 0.78), elbow flexion/extension (r = 0.00, *p* = 1.00), wrist flexion/extension (r = −0.12, *p* = 0.77) and finger flexion/extension (r = −0.28, *p* = 0.46).

In a sub analysis, the relationship between the kinematic metric and the related FMA-UE subsection was explored. The correlation between the FMA-UE arm section and trunk compensation resulted in r = −0.57 (*p* = 0.11). Elbow flexion/extension correlated statistically significantly with the FMA-UE arm subsection with r = 0.68 (*p* = 0.04). The relationship between the FMA-UE wrist subsection and wrist flexion/extension (r = 0.00, *p* = 0.99) as well as between the FMA-UE hand subsection and finger flexion/extension (r = −0.56, *p* = 0.11) was not conclusive.

### 3.5. Relationship between the Selected Joint Range Metrics

Similarly, the relationship between the selected joint range metrics did not result in significant correlations, except for trunk compensation and elbow flexion/extension. A statistically significant correlation was found between the mean trunk compensation and the elbow flexion/extension in the AF with a negative relationship (r = −0.88, *p* = 0.0031) as shown in Table 4. In the NAF statistically significant correlations were found between wrist and finger flexion/extension with strong positive correlations (r = 0.72, *p* = 0.0369).

**Figure 5:**
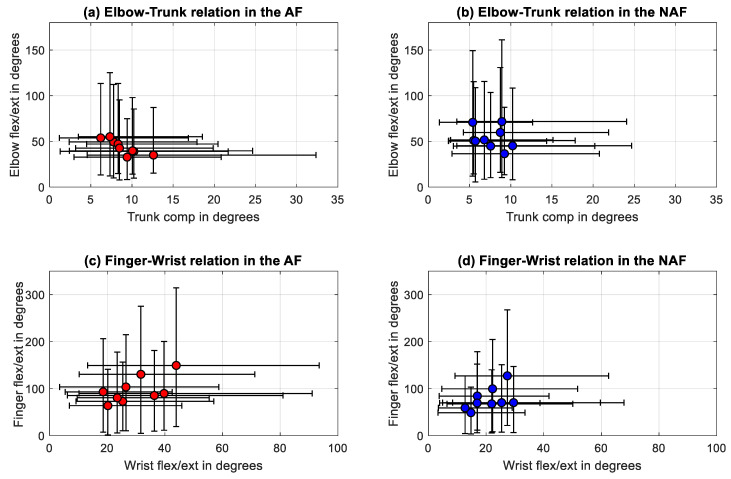
Correlations between (**a**) trunk compensation and elbow flexion/extension in the affected side, (**b**) trunk compensation and elbow flexion/extension in the non-affected side, (**c**) wrist and finger flexion/extension of the affected side, and (**d**) wrist and finger flexion/extension of the non-affected side.

The relationship between the statistically significant correlations between the DOF, elbow flexion/extension joint ranges against trunk compensation and wrist against finger flexion/extension joint ranges were further evaluated by visualizing, as presented in Figure 5. The linear regression line between the trunk and the elbow joint ranges of the AF was defined by *y* = −3.6*x* + 76. Linear regression between the wrist and finger flexion/extension joint ranges was described by *y* = 2.1*x* + 34.

## 4. Discussion

In this pilot study, sensor-based upper limb kinematic measurements of reach-to-grasp and displacement activities executed by chronic stroke subjects were used to examine and relate characteristics of movement impairments and to explore the influences of additional weight loads and mechanical work requirements on the upper limb kinematics. Movement impairments, such as longer movement times and decreased range of motion across the upper limb DOF were found in the affected when compared to the non-affected side, supported by existing literature [23,38]. Besides the evidence for weakness and impaired interjoint coordination in the affected upper limb, illustrated in the consistently decreased shoulder flexion and elbow extension for the whole task execution, this study focused on investigating the expression of pathological coupling between the trunk, elbow, wrist and fingers during object displacement within maximal arm length, as most clearly represented in Table 4. In order to include distally pronounced aspects of movement behavior in the kinematic analysis of object grasping and displacement, the range of motion of the wrist and the fingers’ flexion-extension has been included in the analysis. The increased finger flexion in the AF when compared to the NAF expands on the characterization of the pathological flexor synergy and confirms previous research by Miller et al. [39] and Lan et al. [17] that described and increased difficulty to release the finger flexion with increased arm load. The significant positive correlation between finger and wrist flexion/extension in the non-affected upper limb, as shown in Table 4, could be interpreted as a physiological movement synergy allowing the subject to perform efficient grasp function. Herein, factors impacting the force and mechanical work demands were examined to prove the load-dependent appearance of pathological joint coupling in the upper extremity.

On the level of trunk compensatory movements, increased trunk movements were found in tasks with the affected arm when displacing the heavy block, that could be related to a compensation of weakness in the proximal shoulder muscles or weakness of the trunk muscles themselves. If trunk weakness itself was present in the investigated population, this could account as one explanation for why trunk compensatory movements were also detected in the non-affected side of the data set. It can be assumed that trunk weakness itself would diminish the ability to counterbalance an additional arm weight with either the affected or the non-affected limb. Another explanation for increased trunk movements in the non-affected side could be based on the fact that the NAF arm might deviate from complete healthy movement behavior due to indirect deficits in the non-crossing pathways from the ipsilesional cortex [40]. Nevertheless, these findings are in line with Repnik et al., 2018, who investigated the parameters movement time, smoothness, hand trajectory similarity and trunk stability in stroke subjects and healthy subjects when performing the ARAT and found similarly differences in trunk movements, especially early at movement onset, besides also noting occasional trunk motions above 10° in healthy subjects [41]. These findings suggest that the trunk compensation feature should be further studied with respect to diagnostic sensitivity and specificity to quantify stroke-related upper limb impairments. Apart from trunk compensation, an increase of the object weight showed no significant effects on the features of flexion-extension range of motion of the elbow, wrist, and fingers.

Differences related to the target height factor were detected in all tested features and can be partially explained by the different movement trajectory and positioning of the block object with respect to the hand posture between the top shelf and the table locations. The differences in wrist and finger flexion/extension can in part be explained by differences in hand positioning with respect to the target location, e.g., the hand might be more flexed in the wrist when displacing the block to the top shelf. Nevertheless, the strongest effect of target height was found in the elbow flexion/extension ROM, with a mean increase of elbow range of motion 24.0 ± 5.9 degrees in the top shelf locations when compared to the table target locations. This study finding was surprising, since all four target locations were defined by the maximum arm length to assure the requirement of complete elbow extension at the end of the displacement phase. Furthermore, the increased elbow flexion/extension motion in movements with increased gravity impact stand in contrast to previous research and the hierarchical structure of the synergistic movement patterns [15,26], that presume an increased difficulty of uncoupling elbow flexion from shoulder flexion with increased load and motion. The present study’s findings, contrarily, could suggest that range of motion in elbow extension is increased in target positions that have a larger distance to the subjects’ body center and require increased mechanical work against gravity. Despite the tentativeness of these results and the small study population, these outcomes could open new intervention strategies and should be addressed in future research with larger study samples to investigate possible underlying mechanisms. If the identification of factors that influence the increase or decrease in pathological joint coupling is possible, new intervention approaches would be opened to sustain stroke-related movement impairments. Including gradual decrease or increase of the armload has shown benefits for determining the severity of pathological joint coordination and providing patient-centered interventions, as indicated by Ellis and colleagues [42]. The examination of the influence of task conditions on the selected DOFs support the definition of the task-dependent and dynamic appearance of the pathological flexor synergy [13,17,18]. In the current study, the body of research on task-dependent changes based on planar movement task evaluations were extended to evaluations of reach-to-grasp activities in non-laboratory environments with a close linkage to functional activities of daily life. The fact, that we did not find significant effects of the object weight on the upper limb features, elbow, wrist, and finger flexion-extension except for trunk compensation, might be due to the range of object weight selected, from 100 g to 1 kg. Considering previous research on arm loading during reaching reported a maximum additional load of 50% of the arm weight [18] that would result in about 2 kg for an average person of 80 kg and an arm weight of around 5% of the body weight. Nevertheless, the subjects included in this study showed considerable difficulty in grasping and displacing the 1 kg heavy block.

These findings on movement condition effects on the relevant kinematic features stress the importance of considering task-dependent influences, such as gravitational forces and biomechanical constraints, when assessing and treating stroke-related upper limb impairments. Cortes et al. 2017 studied arm motor control in a planar robotic device and found a non-linear relationship between two-dimensional pointing parameters and scores from clinical scales incorporating antigravity strength demands. The authors suggested that arm motor control plateaus at 5 weeks post-stroke, whereas strength improvement, as measured by clinical scales, continues to improve up to 54 weeks post-stroke [43]. In this regard, it would be interesting to extend these objectives to three-dimensional movement tasks and investigate whether arm motor control, when measured in more complex reach-to-grasp movements by use of less motion impeding measurement systems, follows a similar recovery scheme when compared to 2D arm motor control and clinical scales. The usage of wearable sensing allows movement quality to be tracked in terms of kinematics in a less obstructive and more flexible way.

Another question addressed in this study was the relationship between the kinematic features of the displacement phase and the clinically measured individual impairment level. No clear correlations were found between the kinematic metrics and the FMA-UE, whereas trunk compensation and elbow flexion/extension showed strong correlation with the FMA-UE arm subsection, as well as the correlation between the FMA-UE hand subsection and finger flexion/extension. These findings are in line with existing research [24,44] and support the fact that kinematic parameters are, rather, complementary than redundant to standard clinical scales and potentially add clinically relevant information. The large interquartile ranges in all measured DOF in all study subjects illustrates the large variability in movement execution especially in non-cyclical discrete motions. The negative correlation between trunk compensation and elbow flexion/extension in the movements of the affected limb can be interpreted as an expression of the pathological joint coupling in stroke, where trunk compensation is increased relatively to the lack of active range of motion in the elbow during reaching. The significant positive correlation between the wrist and finger flexion/extension in the non-affected side could account for the appearance of physiological movement synergies during grasping and displacement that is less strong including larger interquartile ranges in both joints and non-significant in the affected side. These results support the use of the selected spatiotemporal features by use of non-laboratory kinematic movement analysis to assess aspects upper limb movement quality and impairments after stroke. Capturing and analyzing the relevant joint ranges during functional activities provides additional complementary information concerning how functional movements are performed and thereby help to overcome limitations of most existing clinical scales. Being able to detect the main aspects of movement quality and impairments allows selecting and monitoring changes in functional outcome and planning interventions that target these aspects. Future research should consider and re-evaluate the outcome features and task considerations presented herein on larger sample sizes to further underpin existing evidence of sufficient validity and reliability for metrics of joint range of motion and trunk displacement [24,44]. Furthermore, analysis of the assessments’ clinimetric properties should be extended to domains sensitivity and specificity for differentiation physiological and pathological movement behavior.

### 4.1. Implementation of the Device and Analysis Methods in Clinical Practice

Wearable devices for assessments of motor function have been an ongoing research direction over the last decade. Portable devices facilitate the setup time and do not require patients to be directed to specific labs for measurements. The presented system potentiates the objective monitoring of the patients’ impairments and provides the therapists an additional and more precise information about the movements’ profile. The collective use of visual observations by the clinician and objectively measured patient movements using a sensing system as proposed in the current study system is intended to be used as means to provide better diagnostic and, thus, better therapy outcomes by providing a more thorough evaluation. Further research should focus on a clinician’s point of view in the usability of the system in the clinic. By instructing therapists on how to use and analyze the distributed measuring system and its output, it is possible to obtain feedback, both from the patient and therapist, on its usability and relevance. In future, and after iterating the development steps of the device and methodology based on the feedback received, objective measurements with these types of system can become the standard for motor function evaluation.

### 4.2. Strength and Limitations

As a main limitation of this pilot study, the small sample size of the study needs to be considered as a factor that suppresses the robustness and degree of reliance of the findings and results presented. However, the investigated sample was homogenic with respect to a limited upper limb capacity, as determined by Hoonhorst et al., 2014, and allowed exploration of the applicability of the multisensory wearable system in the target population at an early device development stage [37]. The study sample included was intended to be able to perform reach-to-grasp and displace movements, which excludes more severely affected subjects. Nevertheless, besides the similar overall upper limb impairments, the included subjects showed reasonable variation in terms of the deficit distribution in the corresponding limb segments, as depicted in Table 2.

The principal idea of combining multiple sensing modalities, such as inertial sensing and other signal quantities, in a wearable system for upper limb kinematic motion analysis was considered as a strength of the device used, as this allows both for simplification or extension of the measurement modalities and enables the conduction of neurophysiological and biomechanical experiments on post-stroke upper limb movements in relatively unrestricted measurement surroundings. The wearable measurement system presented here combined complete kinematic motion analysis of the main DOFs of the upper limb kinematic chain and interaction force measurements at the fingertip, that have shown to be a powerful tool in reach-to-grasp detection and could further inform through measurements of grasp control. Although, we could confirm the application for assessing upper limb movements in chronic stroke subjects in this pilot study, the usability in clinical practice, including set-up, running and analyzing and the selected outcomes, would need to be addressed in future research.

Unfortunately, the force-sensitive resistor sensors used in this study showed limitations in capturing low forces per area and diminished flexibility to adapt to the shape of the finger pad and the grasped object. Therefore, grasp force could not be quantified as an outcome measure apart from the phase segmentation detection. An advanced version of flexible fingerprint sensors, as described in Wolterink et al. [45] is intended to be incorporated in the next generation of this multisensory measurement device. Detecting normal and shear force during grasp can provide further insights into movement control and effectiveness [46]. The combination of kinetic and kinematic measurements would allow to further study grasp control and stroke-related deficits, such as force limitation due to weakness or findings on force overshoot [2]. Effective grasping is undertaken by placing single fingers perpendicularly to the object surface [47]. This could be further explored in subjects after stroke with more adequate kinetic measurements.

Another considerable limitation relates to the systems’ measurement accuracy. Similar to other IMU sensors, the systems’ measurement accuracy depends on a successful sensor and sensor-to-segment calibration, appropriate filtering and fusion algorithms and reliable segment and joint angle definitions [25]. The accuracy of measurements was assured by updates of the global frame orientation definition and the avoidance of unnecessary extra movements prior to each task execution, which lasted not longer than nine seconds.

The detection of phases related to the movement primitives of reaching, object transport and return was feasible by a set of automatic detection algorithms in 47.8% of the affected upper limb data and 52.2% of the non-affected movement data. The observer-based validation of the points for phase discrimination and manual correction of defective time points to differentiate movement phases remain limited to subjective decision-making and time-consuming in processing. The grasping and release point, defined by an increase and decrease of the force profile and/or angular velocity in flexion-extension of the index finger, could show deviations due to inconsistent finger motion and force signals. In particular, the point of object release was difficult to detect when no distal signal peaks were detectable and could be affected by a systematic error if, for example, only maximum elbow extension is used to determine object release, which has to be considered rather as an indirect assumption than a proof of object release. Additionally, periods of transition or “dead time” between the phases need to be considered, as for example at movement start and end, where indifferent minor motions could affect the threshold detection. The application of improved flexible fingertip force sensors would reasonably improve the accuracy and reliability of time-points for phase detection of reach, displacement and return that are in alignment with studies on comparable movement analysis [2,41]. The accurate and time-efficient detection of motion primitive phases of reach-to-grasp activities is a relevant requirement for comparable and repeatable motion analysis of upper limb function.

Finally, we acknowledge that beside movement time and joint range of motion, several other kinematic parameters could have been investigated, such as hand trajectories or smoothness measures to complement the picture of movement quality and impairments. Based on the fact that signal information for the parameter calculation is provided by the system, this could be addressed in future studies using this multisensory measurement device. The data acquired in this study was publically made available for transparent reporting and re-evaluation and extension of the results [48]. To realize the long-term goal of upper limb kinematic assessments in clinical practice, this pilot study investigated metrics that were appropriate to detect and quantify impaired movement behavior after stroke by use of a wearable inertial measurement system. Even though the suggested metrics were derived from well-defined movement tasks, it is reasonable to include these metrics in existing analysis, that have been proven to be useful in the evaluation of non-structured daily-life activities [49,50]. Additionally, considering movement task characteristics and factors influencing the movement behavior were *p* to enable the evaluation of subject-specific motion aspects and assessing the dynamics of the impairments.

## 5. Conclusions

This pilot study demonstrates the applicability of sensor-based kinematic motion analysis of functional reach-to-grasp and displacement movements in chronic stroke subjects with limited upper limb capacity by use of a wearable inertial sensing system. Relevant features to determine upper limb upper limb movement quality were suggested and examined for influences caused by the tested arm, object weight, target height factors and with respect to clinically measured impairment level. Range of motion in trunk displacement, elbow, wrist, and finger flexion-extension showed considerable differences between the AF and the NAF. Effects on metrics for interjoint coordination, as defined by the features, trunk compensation, elbow, wrist, and finger flexion-extension during displacement were found for the factors of an increase in object weight and target height. Hence, the factor’s object weight and target height were suggested to study expressions of the pathological flexor synergy in functional reach-to-grasp movements with different task conditions. The significant correlations between elbow flexion/extension and trunk compensation detected in the affected upper limb support the appearance of pathological joint coupling during object displacement. Range of motion in elbow flexion-extension tended to be lower in the affected side when compared to the non-affected. The finger flexion-extension ROM showed significant differences between the AF and NAF and between the target heights, supporting further evaluation of this feature to quantify distally pronounced aspects of the pathological flexor synergy. These findings support the assessment of kinematic features of reach-to-grasp and displacement movements by use of IMUs and, therefore, help in paving the path towards clinically meaningful and feasible upper limb kinematic assessments in stroke research and clinical practice. The additional investigations on the effect of additional arm load and target height revealed relevant findings in the field of neurophysiology with respect to pathological joint coupling after stroke and highlight important considerations for upper limb kinematic assessments and possible treatment strategies to restore quality of movement in order to regain functionality in activities of daily life.

## Figures and Tables

**Table 1:** Sensor-to-segment calibration protocol.

No	Calibration Position/Movement	Anatomical Axes	
		Left Arm	Right Arm
1	Hand held in pronation flat on the metal box	$z_{h}$, $z_{i}$, $z_{m}$	$z_{h}$, $z_{i}$, $z_{m}$
2	Hand held in sagittal plane with 90° elbow flexion	$-y_{h}$, $-y_{i}$, $-y_{m}$	$y_{h}$, $y_{i}$, $y_{m}$
3	Thumb held flat on the metal box	$z_{t}$	$z_{t}$
4	Thumb held in sagittal plane with the hand in pronation	$y_{t}$	$-y_{t}$
5	Forearm held in pronation along the transversal plane	$z_{la}$	$z_{la}$
6	Forearm motion from supination to pronation in elbow flexion	$x_{la}$	$-x_{la}$
7	Upper arm held parallel to the sagittal plane with 90° elbow flexion	$-x_{ua}$	$-x_{ua}$
8	Shoulder horizontal abduction with 90° elbow flexion	$z_{ua}$	$z_{ua}$
9	Standing straight	$z_{sh}$, $z_{st}$	$z_{sh}$, $z_{st}$
10	Bending forward by hip flexion until around 60°	$y_{sh}$, $y_{st}$	$-y_{sh}$, $-y_{st}$

**Table 2:** Demographic and clinical characteristics of the participants (*n* = 10). Subject S02 was discarded from the data analysis.

Part ID	M/F	Age in Years	Month Since Stroke	Type	Cerebral Artery Territory Affected	Affected Body Side	FMA-UE Total/66	FMA-UE Arm/36	FMA-UE Wrist/10	FMA-UE Hand/14
S01	M	72	73	Isch	PCA	L	35	22	6	10
S02	M	73	30	Isch	MCA	R	33	22	7	9
S03	M	62	181	Hem	(BG)	R	33	26	7	5
S04	F	61	70	Isch	ACA	L	40	22	6	13
S05	M	57	45	Isch	ACA	L	46	31	8	11
S06	F	70	21	Isch	PCA	R	34	22	5	9
S07	F	70	33	Isch	MCA	L	34	22	6	11
S08	M	59	9	Isch	MCA	R	32	16	4	11
S09	F	42	20	Isch	MCA/ICA	R	32	24	3	6
S10	M	42	13	Isch	MCA/PCA	L	28	25	6	2
Median (IQR)		61.5 (57.5–70)	31.5 (20.3–63.8)				33.5 (32.3–34.8)	22 (22–24)	6 (5.3–6.8)	9.5 (6.8–11)
Mean ± SD		60.8 ± 11.4	49.5 ± 51.2				34.7 ± 4.9	23.2 ± 3.8	5.8 ± 1.5	8.7 ± 3.4

Legend: ACA, anterior cerebral artery; (BG), basal ganglia; FMA-UE Total, Fugl–Meyer Assessment of the Upper Extremity; FMA-UE arm, FMA-UE arm subsection; FMA-UE wrist, FMA-UE wrist subsection, FMA-UE hand, FMA-UE hand subsection; Hem, hemorrhage; ICA, internal carotid artery; Isch, ischemic; L, left body side; MCA, middle cerebral artery; M/F, male/female; PCA, posterior cerebral artery; R, right body side.

**Table 3:** Statistical significance of the effects of the independent fixed factors arm, object, and height on the dependent variables of the selected joint range metrics. The factor object including post-hoc pairwise testing between the three levels (BL, BW, BH).

Factor	Trunk Compensation	Elbow Flexion Extension	Wrist Flexion Extension	Finger Flexion Extension
Arm (AF vs. NAF)	0.006 **	0.000 ***	0.000 ***	0.000 ***
Object (BL, BW, BH)	0.022 *	0.146	0.401	0.588
−BL vs. BW	1.000	0.680	1.000	1.000
−BL vs. BH	0.026 *	1.000	0.543	1.000
−BW vs. BH	0.067	0.156	1.000	1.000
Height (Tab vs. Top)	0.006 **	0.000 ***	0.040 *	0.006 **

Legend: *, **, *** indicate statistical significance of *p* < 0.05, *p* < 0.01 and *p* < 0.001, respectively. AF, affected side; BH, heavy block; BL, light block; BW, wooden block; NAF, non-affected side; Tab, table target position; Top, top location.

**Table 4:** Confusion matrix of the Spearman rank correlation coefficients between the selected joint range metrics of the AF and the NAF side.

AF	Trunk Comp	Elbow Flex/Ext	Wrist Flex/Ext	Finger Flex/Ext	NAF	Trunk Comp	Elbow Flex/Ext	Wrist Flex/Ext	Finger Flex/Ext
**Trunk Comp**	1.00	−0.88 **	0.05	0.10	**Trunk Comp**	1.00	−0.35	0.08	0.17
**Elbow Flex/Ext**	.	1.00	−0.32	−0.20	**Elbow Flex/Ext**	.	1.00	0.35	−0.03
**Wrist Flex/Ext**	.	.	1.00	0.53	**Wrist Flex/Ext**	.	.	1.00	0.72 *
**Finger Flex/Ext**	.	.	.	1.00	**Finger Flex/Ext**	.	.	.	1.00

* indicates the statistical significance of the correlation with *p* < 0.05 and ** indicating statistical significance of the correlation with *p* < 0.01.

**Figure A1:**
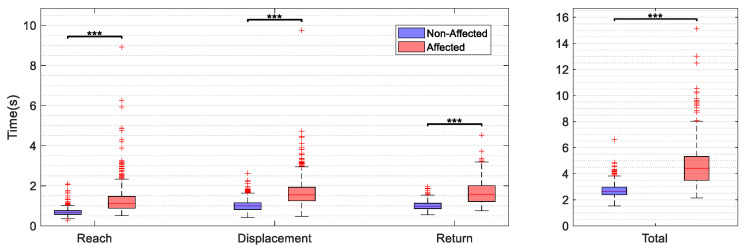
Movement time in seconds represented for the phases of reach, displacement and return and the total movement time. Data include all trials, object weights and target positions for the affected side (AF) and non-affected side (NAF).

## Appendix A

As a proof of concept validation, differences between the affected and non-affected sides in movement time and range of motion of the main DOFs were tested for statistically significant differences between the dependent groups by use of the Wilcoxon signed rank test, as illustrated in Figure A1 and Table A1. For movement time, all trial of the affected and non-affected sides were considered, whereas range of motion was compared between affected and non-affected sides, evaluation all trials separately for each target location.

**Table A1:** Differences in range of motion (AF vs. NAF) for the main upper limb degrees of freedom per target location.

Target Side	Trunk Comp	Shoulder Flex/Ext	Shoulder Abd/Add	Elbow Flex/Ext	Forearm Sup/Pro	Wrist Flex/Ext	Thumb Flex/Ext	Index Flex/Ext	Middle Flex/Ext
Tab 1 AF	17.37 ± 5.81	35.21 ± 10.65	24.02 ± 9.60	48.47 ± 18.11	43.98 ± 22.90	42.68 ± 15.83	82.48 ± 33.57	98.64 ± 37.73	101.19 ± 31.54
Tab 1 NAF	16.47 ± 6.10	49.03 ± 9.39	25.38 ± 10.69	63.62 ± 14.18	38.00 ± 10.53	37.19 ± 10.44	98.46 ± 39.26	76.13 ± 31.54	93.13 ± 27.47
*p*-value	0.149	***	0.446	***	***	*	***	***	***
Tab 2 AF	13.18 ± 7.63	25.93 ± 11.42	35.66 ± 9.05	51.92 ± 12.64	49.12 ± 23.24	45.00 ± 14.47	81.11 ± 32.19	95.65 ± 40.08	97.38 ± 32.33
Tab 2 NAF	8.17 ± 4.80	35.83 ± 10.00	33.71 ± 7.87	65.91 ± 14.62	38.00 ± 10.53	40.09 ± 13.22	97.48 ± 40.74	74.25 ± 32.03	90.64 ± 28.58
*p*-value	***	***	*	***	***	*	**	***	***
Top 3 AF	15.41 ± 5.06	47.36 ± 11.53	31.41 ± 10.41	58.20 ± 17.34	60.63 ± 30.96	48.47 ± 18.09	85.89 ± 38.89	107.74 ± 41.14	103.75 ± 28.04
Top 3 NAF	15.63 ± 6.08	60.21 ± 12.12	29.71 ± 11.05	71.87 ± 13.12	45.78 ± 18.03	44.38 ± 14.00	100.45 ± 40.75	85.54 ± 30.15	98.94 ± 28.34
*p*-value	0.287	***	0.116	***	***	0.264	***	***	*
Top 4 AF	16.16 ± 9.03	33.41 ± 11.08	45.44 ± 10.98	65.36 ± 12.89	72.55 ± 27.12	51.06 ± 19.96	86.28 ± 38.53	110.81 ± 39.35	103.24 ± 32.21
Top 4 NAF	9.69 ± 3.80	41.23 ± 12.80	39.63 ± 10.44	77.44 ± 12.55	63.66 ± 16.27	46.88 ± 14.23	100.56 ± 44.34	82.13 ± 31.31	99.67 ± 29.52
*p*-value	***	***	***	***	*	0.060	*	***	0.105

Means and standard deviations (±) are presented for each DOF and target location (Tab 1, Tab 2, Top 3, Top 4) *n* the affected (AF) and non-affected side (NAF). Statistical significance of *p* < 0.05 is indicated by *, of *p* < 0.01 by ** and of *p* < 0.001 by ***. Legend: Abd/Add, abduction/adduction; Flex/Ext, flexion/extension; Sup/Pro, supination/pronation; Trunk Comp, trunk compensation.

## References

[1] Jones L.A., Lederman S.J. (2006). Human Hand Function.

[2] Parry R., Soria S.M., Pradat-Diehl P., Marchand-Pauvert V., Jarrassé N., Roby-Brami A. (2019). Effects of Hand Configuration on the Grasping, Holding, and Placement of an Instrumented Object in Patients With Hemiparesis. Front. Neurol..

[3] Feix T., Romero J., Schmiedmayer H.-B., Dollar A.M., Kragic D. (2016). The GRASP Taxonomy of Human Grasp Types. IEEE Trans. Hum. Mach. Syst..

[4] Bernstein N.A. (1967). The Coordination and Regulation of Movements.

[5] Jeannerod M. (1994). The representing brain: Neural correlates of motor intention and imagery. Behav. Brain Sci..

[6] Ekstrand E., Rylander L., Lexell J., Brogårdh C. (2016). Perceived ability to perform daily hand activities after stroke and associated factors: A cross-sectional study. BMC Neurol..

[7] Vos T., Allen C., Arora M., Barber R.M., Bhutta Z.A., Brown A., Carter A., Casey D.C., Charlson F.J., Chen A.Z. (2016). Global, regional, and national incidence, prevalence, and years lived with disability for 310 diseases and injuries, 1990–2015: A systematic analysis for the Global Burden of Disease Study 2015. Lancet.

[8] Sacco R.L., Kasner S.E., Broderick J.P., Caplan L.R., Connors J., Culebras A., Elkind M.S., George M.G., Hamdan A.D., Higashida R.T. (2013). An Updated Definition of Stroke for the 21st Century: A statement for healthcare professionals from the American Heart Association/ American Stroke Association. Stroke.

[9] Byblow W.D., Stinear C.M., Barber P.A., Petoe M.A., Ackerley S.J. (2015). Proportional recovery after stroke depends on corticomotor integrity. Ann. Neurol..

[10] Stinear C.M., Barber P.A., Smale P.R., Coxon J.P., Fleming M.K., Byblow W.D. (2007). Functional potential in chronic stroke patients depends on corticospinal tract integrity. Brain.

[11] Raghavan P. (2015). Upper Limb Motor Impairment After Stroke. Phys. Med. Rehabil. Clin. N. Am..

[12] Santello M., Lang C.E. (2015). Are Movement Disorders and Sensorimotor Injuries Pathologic Synergies? When Normal Multi-Joint Movement Synergies Become Pathologic. Front. Hum. Neurosci..

[13] Sukal-Moulton T., Ellis M.D., Dewald J.P. (2007). Shoulder abduction-induced reductions in reaching work area following hemiparetic stroke: Neuroscientific implications. Exp. Brain Res..

[14] Levin M.F. (1996). Interjoint coordination during pointing movements is disrupted in spastic hemiparesis. Brain.

[15] Twitchell T.E. (1951). The Restoration of Motor Function Following Hemiplegia in Man. Brain.

[16] Ellis M.D., Sukal-Moulton T., Dewald J.P.A. (2009). Progressive shoulder abduction loading is a crucial element of arm rehabilitation in chronic stroke. Neurorehabil. Neural Repair.

[17] Lan Y., Yao J., Dewald J.P. (2017). The Impact of Shoulder Abduction Loading on Volitional Hand Opening and Grasping in Chronic Hemiparetic Stroke. Neurorehabil. Neural Repair.

[18] Ellis M.D., Lan Y., Yao J., Dewald J.P. (2016). Robotic quantification of upper extremity loss of independent joint control or flexion synergy in individuals with hemiparetic stroke: A review of paradigms addressing the effects of shoulder abduction loading. J. Neuroeng. Rehabil..

[19] Cirstea C.M., Levin M.F. (2000). Compensatory strategies for reaching in stroke. Brain.

[20] Kwakkel G., Lannin N.A., Borschmann K., English C., Ali M., Churilov L., Saposnik G., Winstein C., van Wegen E.E.H., Wolf S.L. (2017). Standardized measurement of sensorimotor recovery in stroke trials: Consensus-based core recommendations from the Stroke Recovery and Rehabilitation Roundtable. Int. J. Stroke.

[21] Platz T., Pinkowski C., van Wijck F., Kim I.-H., di Bella P., Johnson G. (2005). Reliability and validity of arm function assessment with standardized guidelines for the Fugl-Meyer Test, Action Research Arm Test and Box and Block Test: A multicentre study. Clin. Rehabil..

[22] Collins K.C., Kennedy N.C., Clark A., Pomeroy V.M. (2018). Getting a kinematic handle on reach-to-grasp: A meta-analysis. Physiotherapy.

[23] Murphy M.A., Häger C.K. (2015). Kinematic analysis of the upper extremity after stroke—How far have we reached and what have we grasped?. Phys. Ther. Rev..

[24] Schwarz A., Kanzler C.M., Lambercy O., Luft A.R., Veerbeek J.M. (2019). Systematic Review on Kinematic Assessments of Upper Limb Movements After Stroke. Stroke.

[25] Walmsley C.P., Williams S.A., Grisbrook T.L., Elliott C., Imms C., Campbell A. (2018). Measurement of Upper Limb Range of Motion Using Wearable Sensors: A Systematic Review. Sports Med. Open.

[26] Fugl-Meyer A.R., Jääskö L., Leyman I., Olsson S., Steglind S. (1975). The post-stroke hemiplegic patient. 1. a method for evaluation of physical performance. Scand. J. Rehabil. Med..

[27] Kortier H.G., Sluiter V.I., Roetenberg D., Veltink P.H. (2014). Assessment of hand kinematics using inertial and magnetic sensors. J. Neuroeng. Rehabil..

[28] van den Noort J.C., Kortier H.G., van Beek N., Veeger D.H.E.J., Veltink P.H. (2016). Measuring 3D Hand and Finger Kinematics—A Comparison between Inertial Sensing and an Opto-Electronic Marker System. PLoS ONE.

[29] Brodie M.A., Walmsley A., Page W., Brodie M.A. (2008). The static accuracy and calibration of inertial measurement units for 3D orientation. Comput. Methods Biomech. Biomed. Eng..

[30] Luinge H., Veltink P.H., Baten C.T.M. (2007). Ambulatory measurement of arm orientation. J. Biomech..

[31] Ricci L., Formica D., Sparaci L., Lasorsa F.R., Taffoni F., Tamilia E., Guglielmelli E. (2014). A New Calibration Methodology for Thorax and Upper Limbs Motion Capture in Children Using Magneto and Inertial Sensors. Sensors.

[32] Kong W., Sessa S., Zecca M., Takanishi A. (2016). Anatomical Calibration through Post-Processing of Standard Motion Tests Data. Sensors.

[33] Madgwick S.O.H., Harrison A.J.L., Vaidyanathan R. Estimation of IMU and MARG orientation using a gradient descent algorithm. Proceedings of the 2011 IEEE International Conference on Rehabilitation Robotics.

[34] Kirking B., El-Gohary M., Kwon Y. (2016). The feasibility of shoulder motion tracking during activities of daily living using inertial measurement units. Gait Posture.

[35] Lyle R.C. (1981). A performance test for assessment of upper limb function in physical rehabilitation treatment and research. Int. J. Rehabil. Res..

[36] de Vries J., van Ommeren A., Prange-Lasonder G., Rietman J., Veltink P.H. (2018). Detection of the intention to grasp during reach movements. J. Rehabil. Assist. Technol. Eng..

[37] Hoonhorst M.H., Nijland R.H., van der Berg J.S., Emmelot C.H., Kollen B.J., Kwakkel G. (2015). How Do Fugl-Meyer Arm Motor Scores Relate to Dexterity According to the Action Research Arm Test at 6 Months Poststroke?. Arch. Phys. Med. Rehabil..

[38] van Kordelaar J., van Wegen E.E.H., Kwakkel G. (2012). Unraveling the interaction between pathological upper limb synergies and compensatory trunk movements during reach-to-grasp after stroke: A cross-sectional study. Exp. Brain Res..

[39] Miller J.C., Dewald J.P.A. (2012). Involuntary paretic wrist/finger flexion forces and EMG increase with shoulder abduction load in individuals with chronic stroke. Clin. Neurophysiol..

[40] Nowak D.A., Grefkes C., Dafotakis M., Eickhoff S., Küst J., Karbe H., Fink G.R. (2008). Effects of Low-Frequency Repetitive Transcranial Magnetic Stimulation of the Contralesional Primary Motor Cortex on Movement Kinematics and Neural Activity in Subcortical Stroke. Arch. Neurol..

[41] Repnik E., Puh U., Goljar N., Munih M., Mihelj M. (2018). Using Inertial Measurement Units and Electromyography to Quantify Movement during Action Research Arm Test Execution. Sensors.

[42] Ellis M.D., Carmona C., Drogos J.M., Traxel S., Dewald J.P. Progressive abduction loading therapy targeting flexion synergy to regain reaching function in chronic stroke: Preliminary results from an RCT. Proceedings of the 2016 38th Annual International Conference of the IEEE Engineering in Medicine and Biology Society (EMBC).

[43] Cortes J.C., Goldsmith J., Harran M.D., Xu J., Kim N., Schambra H.M., Luft A.R., Celnik P., Krakauer J.W., Kitago T. (2017). A Short and Distinct Time Window for Recovery of Arm Motor Control Early After Stroke Revealed With a Global Measure of Trajectory Kinematics. Neurorehabil. Neural Repair.

[44] Kanzler C.M., Rinderknecht M.D., Schwarz A., Lamers I., Gagnon C., Held J., Feys P., Luft A.R., Gassert R., Lambercy O. (2020). A data-driven framework for selecting and validating digital health metrics: Use-case in neurological sensorimotor impairments. NPJ Digit. Med..

[45] Wolterink G.J.W., Sanders R.G.P., Krijnen G. Thin, Flexible, Capacitive Force Sensors Based on Anisotropy in 3D-Printed Structures. Proceedings of the 2018 IEEE Sensors Applications Symposium (SAS).

[46] Nowak D.A., Rosenkranz K., Topka H., Rothwell J. (2005). Disturbances of grip force behaviour in focal hand dystonia: Evidence for a generalised impairment of sensory-motor integration?. J. Neurol. Neurosurg. Psychiatry.

[47] Cuijpers R.H., Smeets J.B., Brenner E. (2004). On the Relation Between Object Shape and Grasping Kinematics. J. Neurophysiol..

[48] Schwarz A., Bhagubai M.M.C., Wolterink G., Held J.P.O., Luft A.R., Veltink P.H. (2020). Kinematics of reach-to-grasp and displacement after stroke [Data set]. https://zenodo.org/record/3930752#.X0EGBjVCRhE.

[49] van Meulen F.B., Klaassen B., Held J., Reenalda J., Buurke J.H., van Beijnum B.F., Luft A., Veltink P. (2016). Objective Evaluation of the Quality of Movement in Daily Life after Stroke. Front. Bioeng. Biotechnol..

[50] Held J.P.O., Klaassen B., Eenhoorn A., van Beijnum B.-J.F., Buurke J.H., Veltink P., Luft A.R. (2018). Inertial Sensor Measurements of Upper-Limb Kinematics in Stroke Patients in Clinic and Home Environment. Front. Bioeng. Biotechnol..

